# Anti-inflammatory effects of proprotein convertase subtilisin/kexin 9 inhibitor therapy in the early phase of acute myocardial infarction

**DOI:** 10.1007/s00380-024-02473-8

**Published:** 2024-10-05

**Authors:** Tomohiro Shimizu, Tetsuji Morishita, Hiroyasu Uzui, Yusuke Sato, Tatsuhiro Kataoka, Machiko Miyoshi, Junya Yamaguchi, Yuichiro Shiomi, Hiroyuki Ikeda, Naoto Tama, Kanae Hasegawa, Kentaro Ishida, Hiroshi Tada

**Affiliations:** 1 Department of Cardiovascular Medicine, Faculty of Medical Sciences, University of Fukui, 23-3 Shimoaizuki, Matsuoka Eiheiji-cho, Yoshida-gun, Fukui, 910-1193 Japan; 2 Department of Internal Medicine, Matsunami General Hospital, Gifu, 501-6062 Japan; 3 Department of Clinical Nursing, Faculty of Medical Sciences, University of Fukui, Fukui, 910-1193 Japan

**Keywords:** Proprotein convertase subtilisin/kexin 9 inhibitor, Tumor necrosis factor-α, l-arginine/asymmetric-dimethylarginine, Acute myocardial infarction

## Abstract

This study examined the anti-inflammatory and endothelial function-enhancing effects of proprotein convertase subtilisin/kexin 9 (PCSK9) inhibitor therapy in the early phase after acute myocardial infarction (AMI) by assessing changes in tumor necrosis factor-α (TNF-α) levels and the l-arginine/asymmetric-dimethylarginine (ADMA) ratio. This retrospective, single-center cohort study included patients who underwent successful timely primary percutaneous coronary intervention (PCI) for first-onset AMI between September 2017 and March 2018. The PCSK9 inhibitor group comprised patients who received 75 mg alirocumab up to 7 days after AMI, while the standard therapy group comprised patients who did not. We evaluated the change in TNF-α levels and the l-arginine/ADMA ratio at the time of hospital admission and prior to discharge. PCSK9 inhibitor therapy in the early phase after AMI suppressed TNF-α levels (standard therapy group, 1.64 ± 2.14 pg/mL vs. PCSK9 inhibitor group, 0.26 ± 0.33 pg/mL; *p* = 0.033) and increased the L-arginine/ADMA ratio (standard therapy group, − 13.0 ± 39.7 vs. PCSK9 inhibitor group, 23.2 ± 39.7; *p* = 0.042). Upon multiple regression analysis adjusted for sex, age, and peak creatine kinase levels, PCSK9 inhibitor therapy was associated with TNF-α suppression (*p* = 0.025; *β* = − 0.235, 95% confidence interval [CI], − 0.436 to − 0.033). The L-arginine/ADMA ratio was also analyzed using multiple regression, adjusted for sex, age, peak creatine kinase levels, and smoking, showing a significant improvement in the ratio (*p* = 0.018; *β* = 41.913, 95% CI, 10.337−73.491). Moreover, a weak negative correlation was suggested between the change in TNF-α levels and the change in l-arginine/ADMA ratio (*r* = − 0.393, *p* = 0.058). PCSK9 inhibitor therapy in the early phase after AMI suppresses TNF-α levels and improves the l-arginine/ADMA ratio, potentially indicating anti-inflammatory and endothelial function-enhancing effects.

## Introduction

Despite advances in reperfusion therapy, such as percutaneous coronary intervention (PCI), heart failure following myocardial infarction remains a significant clinical concern [1]. To prevent the progression of heart failure in patients with myocardial infarction, it is crucial to reduce cardiomyocyte death, although no established treatment currently exists to rescue cardiomyocytes in these patients [2]. Coronary artery revascularization-induced myocardial microcirculatory dysfunction is a primary factor contributing to heart failure following acute myocardial infarction (AMI) [3]. Several factors, such as endothelial dysfunction and inflammation, are implicated in the development of microvascular dysfunction after AMI [4, 5]. Myocardial ischemic reperfusion injury induces microvascular injury and furthers myocardial necrosis, leading to an increase in tumor necrosis factor-α (TNF-α) levels [6]. The L-arginine/asymmetric dimethylarginine (ADMA) ratio has been found to decrease not only in myocardial infarction but also in cases of microvascular dysfunction [7–9]. Recent studies indicate that reperfusion injury increases proprotein convertase subtilisin/kexin 9 (PCSK9) expression in reperfused heart tissues, which is involved in the creation of scar formation and the development of cardiac dysfunction. Moreover, the use of PCSK9 inhibitors prior to myocardial infarction has been shown to suppress PCSK9 expression, decelerate the process of autophagy, and mitigate myocardial ischemia–reperfusion injury [10].

PCSK9 inhibitors have both lipid-lowering and anti-inflammatory properties [11]. However, the impact of PCSK9 inhibitors on inflammation and endothelial dysfunction in the early phase of AMI remains to be elucidated. Currently, there is no established treatment to suppress inflammation and endothelial dysfunction following AMI. Therefore, this study aimed to investigate the potential of PCSK9 inhibitor therapy to alleviate inflammation and endothelial dysfunction in the early phase of AMI by measuring TNF-α levels and the L-arginine/ADMA ratio.

## Materials and methods

### Study participants and design

This retrospective, single-center cohort study analyzed data from the Department of Cardiovascular Medicine registry database of the University of Fukui. We included patients who underwent successful primary PCI within 24 h for first-onset AMI between September 2017 and March 2018. We excluded patients with malignancy or infection, recipients of inotropic therapy or mechanical support after cardiogenic shock, patients without a history of prior standard therapy, such as dual anti-platelet and statin therapy, and patients lacking blood sample data. Guidelines in Japan recommend administering the maximum tolerable dose of statins from the early stages of AMI [12]. However, the maximum tolerable doses of statins are lower in Japanese patients compared to those in other countries [13]. Based on these factors, we defined “standard statin therapy” in this study as the maximum tolerable dose determined by the attending physician. Patients with a history of prior statin therapy and low-density lipoprotein cholesterol (LDL-C) levels > 100 mg/dL were administered PCSK9 inhibitor therapy (alirocumab 75 mg) during hospitalization. The decision to administer PCSK9 inhibitor therapy was made by the attending physician, who determined that it was necessary for patients with high LDL-C levels. Blood samples were collected during hospitalization and prior to discharge. AMI was defined as an increase in cardiac biomarkers, such as troponin I and creatine kinase (CK)-MB, accompanied by either ischemic symptoms or the presence of significant new ST-T changes or pathologic Q-waves on the electrocardiogram. AMI was categorized into two types: ST-segment elevation myocardial infarction (STEMI), characterized by new ST segment elevation at the J point in at least two contiguous leads with cut-off points ≥ 0.2 mV in leads V_1_, V_2_, or V_3_ and ≥ 0.1 mV in other leads, or non-ST-segment elevation myocardial infarction (NSTEMI), characterized by new ST segment depression or T-wave abnormalities, such as symmetric T-wave inversions ≥ 1 mm. All patients with STEMI were classified as Killip class I, characterized by the absence of any clinical signs of heart failure. The thrombolysis in myocardial infarction (TIMI) flow grade was classified as 0 (no perfusion), 1 (penetration without perfusion), 2 (partial perfusion), and 3 (complete perfusion) [14]. Hypertension was defined as systolic blood pressure ≥ 140 mmHg, diastolic blood pressure ≥ 90 mmHg, or current use of antihypertensive medication. Left-ventricular ejection fraction was calculated using the modified Simpson method. Blood samples to assess diabetes mellitus and dyslipidemia were collected on admission. Diabetes mellitus was defined as hemoglobin A1c ≥ 6.5% or current use of antidiabetic medications. Dyslipidemia was defined as LDL-C ≥ 140 mg/dL, high-density lipoprotein cholesterol (HDL-C) < 40 mg/dL, non-HDL-C ≥ 170 mg/dL, triglycerides ≥ 150 mg/dL, or current use of statins. The investigation conformed with the principles outlined in the Declaration of Helsinki. This study was approved by the University of Fukui Ethics Committee. Because of the retrospective nature of this study, the requirement for obtaining patients’ informed consent was waived when collecting these administrative data. The details of this study were made publicly available through an opt-out process, with patients being clearly informed of their right to decline participation. The control group comprised patients who underwent a certain treatment technique; the follow-up outcomes were documented in the Universal Hospital Medical Information Network Clinical Trials Registry (UMIN 000052960).

### Laboratory analysis

Whole blood was drawn from either a forearm or femoral vein after overnight fasting and immediately placed on ice. Plasma and serum were separated by centrifugation within 30 min, and the samples were subsequently frozen and stored at − 80 °C until analysis. The TNF-α serum levels were determined using an enzyme-linked immunosorbent assay (Quantikine HS Human TNF-α Immunoassay; R&D Systems, Minneapolis, MN, USA). The serum concentrations of ADMA and L-arginine were determined using high-performance liquid chromatography and precolumn derivatization high-performance liquid chromatography/electrospray mass spectrometry, respectively (Fujifilm Wako Pure Chemical, Osaka, Japan) [15, 16]. We measured peak CK levels by collecting blood samples at baseline and 6, 12, 18, and 24 h after PCI.

### Statistical analysis

Categorical variables are expressed as frequencies and percentages, while the median is used for parameters with non-normal distributions, and the mean ± standard deviation is reported for variables with normal distributions. Differences between categorical variables were assessed using either the Chi-square test or Fisher’s exact test. The correlation between continuous variables was determined using Spearman’s correlation. The change in TNF-α levels was calculated as the difference between TNF-α levels at discharge and admission. Similarly, the change in the L-arginine/ADMA ratio was calculated as the difference between the L-arginine/ADMA ratio at discharge and admission. The Mann−Whitney U test was used to analyze the changes in TNF-α levels and the L-arginine/ADMA ratio. To assess treatment efficacy, multiple linear regression analysis was performed with changes in TNF-α levels as the dependent variable and PCSK9 inhibition, age, sex, and peak CK levels, which correlated with the severity of AMI, as independent variables [17]. Multiple linear regression analysis was performed for the L-arginine/ADMA ratio, with smoking, age, sex, and peak CK levels as independent variables, as the ratio may be influenced by smoking [18]. The changes in TNF-α levels were not normally distributed, as indicated by the Q–Q plot, prompting us to apply a log transformation to meet the assumptions of the regression analysis. The regression coefficients were estimated along with their 95% confidence intervals (CIs). *p* values < 0.05 were considered statistically significant. All statistical analyses were performed using R version 4.2.2 (The R Foundation for Statistical Computing, Vienna, Austria).

## Results

**Fig. 1:**
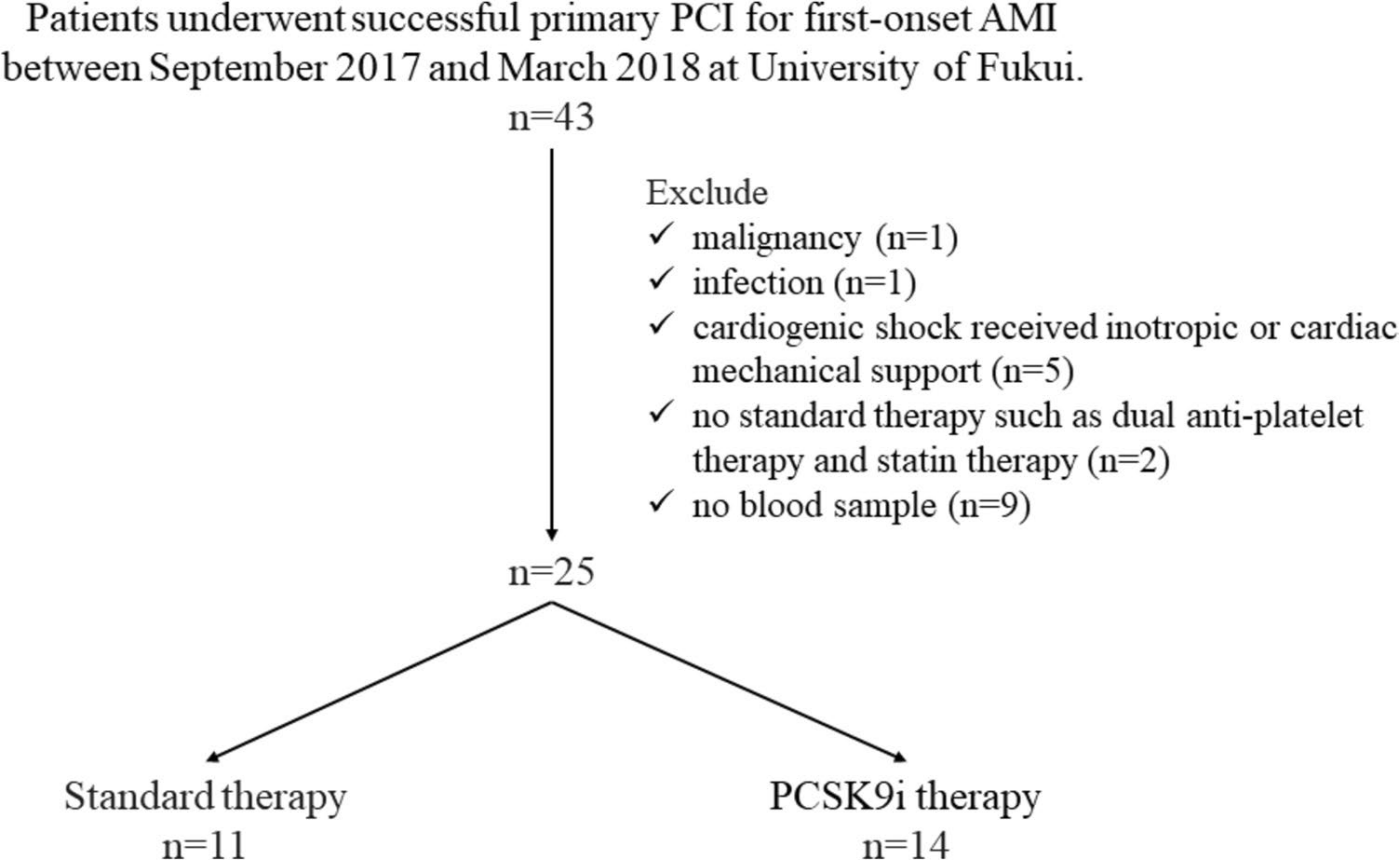
The eligibility criteria of this study. PCSK9i, proprotein convertase subtilisin/kexin 9 inhibitor therapy; PCI, percutaneous coronary intervention; AMI, acute myocardial infarction

A total of 43 patients were enrolled in this study. Among these, 18 patients were excluded due to malignancy (*n* = 1), infection (*n* = 1), cardiogenic shock management with inotropic therapy or mechanical support (*n* = 5), no history of prior standard therapy (*n* = 2), and lack of blood sample data (*n* = 9). Overall, 25 patients were included in this study, with 11 in the standard therapy group and 14 in the PCSK9 inhibitor group (Fig. 1).

### Patient characteristics

No significant differences were observed between the two groups in terms of baseline characteristics, such as age, sex, weight, body mass index, and history of hypertension, dyslipidemia, diabetes mellitus, chronic kidney disease, and smoking. Similarly, no differences were found in AMI type (NSTEMI or STEMI), culprit vessel (left anterior descending artery [LAD] or non-LAD), TIMI flow grade, peak CK levels, ejection fraction (as assessed by echocardiography performed after primary PCI), and LDL-C or HDL-C levels at admission. There was no significant difference in door-to-balloon time between the two groups of patients with STEMI (Table 1). All patients in the PCSK9 inhibitor therapy group were administered alirocumab 75 mg within 2.8 ± 2.0 days after PCI.

**Table 1:** Patients’ characteristics

	Standard therapy (*n* = 11)	PCSK9i therapy (*n* = 14)	*p* value
Age (year)	66.5 ± 10.1	66.5 ± 11.3	0.99
Sex (male), n (%)	11 (100)	10 (71.4)	0.17
Body weight (kg)	59.6 (11.2)	64.3 (14.2)	0.38
BMI (kg/m^2^)	21.8 ± 4.1	24.3 ± 3.7	0.12
Hypertension, n (%)	6 (54.5)	12 (85.7)	0.20
Dyslipidemia, n (%)	11 (100)	14 (100)	
Diabetes mellitus, n (%)	5 (45.5)	4 (28.6)	0.65
Chronic kidney disease, n (%)	2 (18.2)	2 (14.3)	> 0.99
Smoking history, n (%)	9 (81.8)	8 (57.1)	0.38
STEMI, n (%)	8 (72.7)	8 (57.1)	0.70
Door-to-balloon time (min)	97.3 ± 53.4	115.0 ± 60.8	0.40
Culprit vessel (LAD), n (%)	2 (18.2)	5 (35.7)	0.60
TIMI flow grade 3	10 (90.9)	10 (71.4)	0.48
maximal CK (U/I)	2414.6 ± 2254.6	2164.7 ± 3342.4	0.71
Ejection fraction (%)	54.5 (5.4)	56.8 (10.4)	0.53
LDL-C (mg/dL)	102.4 ± 47.2	117.3 ± 18.0	0.29
HDL-C (mg/dL)	44.3 ± 8.0	48.5 ± 8.7	0.23

*BMI* body mass index; *STEMI* ST-segment elevation myocardial infarction; *LAD* left anterior descending artery; *TIMI flow grade* Thrombolysis in myocardial infarction flow grade, *maximal CK* maximal creatine kinase; *LDL-C* low-density lipoprotein cholesterol; *HDL-C* high-density lipoprotein cholesterol

### TNF-α levels and L-arginine/ADMA ratio

No significant differences were observed in blood sampling timing between the two groups. The first sampling occurred at 0.8 ± 0.9 days in the standard therapy group and 1.2 ± 1.2 days in the PCSK9 inhibitor group (*p* = 0.365). The second sampling took place at 16.7 ± 4.5 days in the standard therapy group and 15.4 ± 5.8 days in the PCSK9 inhibitor therapy group (*p* = 0.562).

**Fig. 2:**
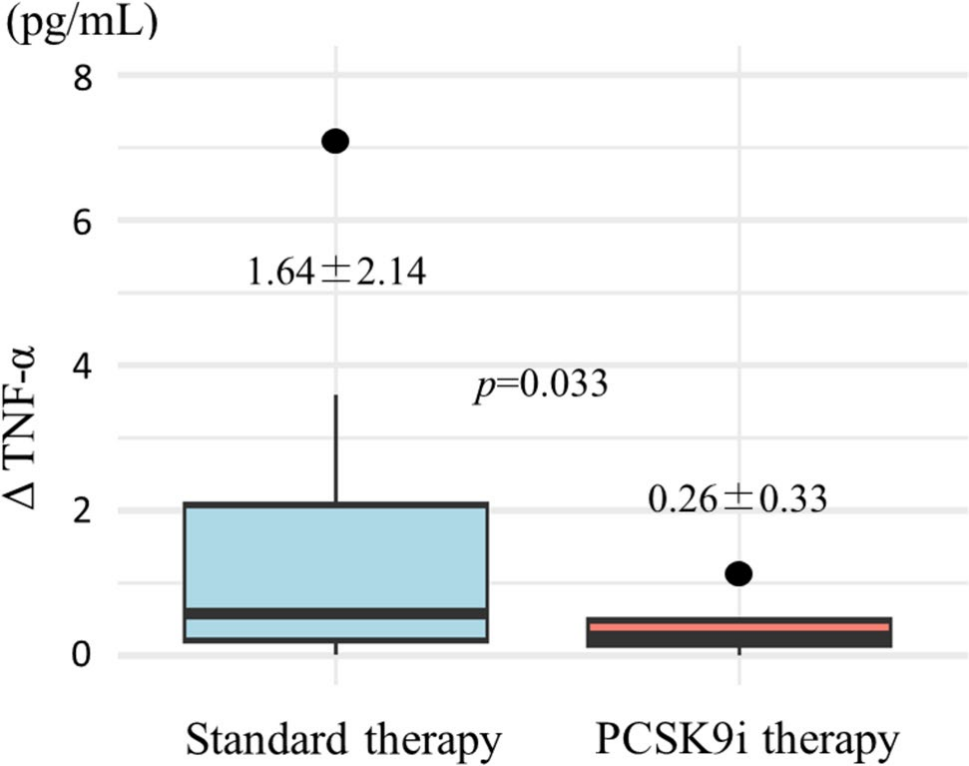
Changes in tumor necrosis factor-α (TNF-α) levels (Δ TNF-α) for both the standard therapy and PCSK9 inhibitor (PCSK9i) groups between admission and discharge. Each boxplot illustrates the interquartile range (IQR) of the changes in TNF-α levels, with the median value highlighted. Whiskers extend from the box to the furthest data points that are within 1.5 times the IQR, denoting variability outside the central 50% of the data. Points beyond the whiskers are outliers, showing extreme TNF-α changes

No significant difference in TNF-α levels was observed at the time of admission, with the standard therapy group at 0.96 ± 0.74 pg/mL and the PCSK9 inhibitor group at 0.95 ± 0.57 pg/mL (*p* = 0.975). Although TNF-α levels increased prior to discharge, the PCSK9 inhibitor group exhibited lower TNF-α levels than the standard therapy group. The change in TNF-α levels, calculated as the difference between TNF-α levels at discharge and admission, was 1.64 ± 2.14 pg/mL and 0.26 ± 0.33 pg/mL in the standard therapy PCSK9 inhibitor groups, respectively (*p* = 0.033) (Fig. 2). In a multiple linear regression analysis adjusted for sex, age, and peak CK levels, TNF-α was also found to be suppressed in the PCSK9 inhibitor group (*p* = 0.025; *β* = − 0.235, 95% CI, − 0.436 to − 0.033) (Table 2).

**Table 2:** Multiple regression analysis of the log-transformed change in TNF-α levels

	Beta	Std. error	95% CI	*t*	*p* value
Constant	0.101	0.314	− 0.555 to 0.756	0.320	0.752
Sex (male)	− 0.010	0.136	− 0.294 to 0.273	− 0.075	0.941
Age (year)	0.003	0.004	− 0.005 to 0.012	0.820	0.422
maximal CK (U/I)	0.0002	0.002	− 0.003 to 0.004	0.097	0.924
PCSK9i therapy	− 0.235	0.097	− 0.436 to − 0.033	− 2.430	0.025

The change in TNF-α levels was defined as the difference between TNF-α levels at discharge and admission. In a multiple linear regression analysis, with the change in TNF-α levels as the dependent variable and sex, age, maximal CK, and PCSK9i therapy as independent variables, PCSK9i therapy significantly reduced TNF-α levels

*maximal CK* maximal creatine kinase; *PCSK9i therapy* proprotein convertase subtilisin kexin 9 inhibitor therapy; *TNF-α* tumor necrosis factor-α; *CI* confidence interval

**Fig. 3:**
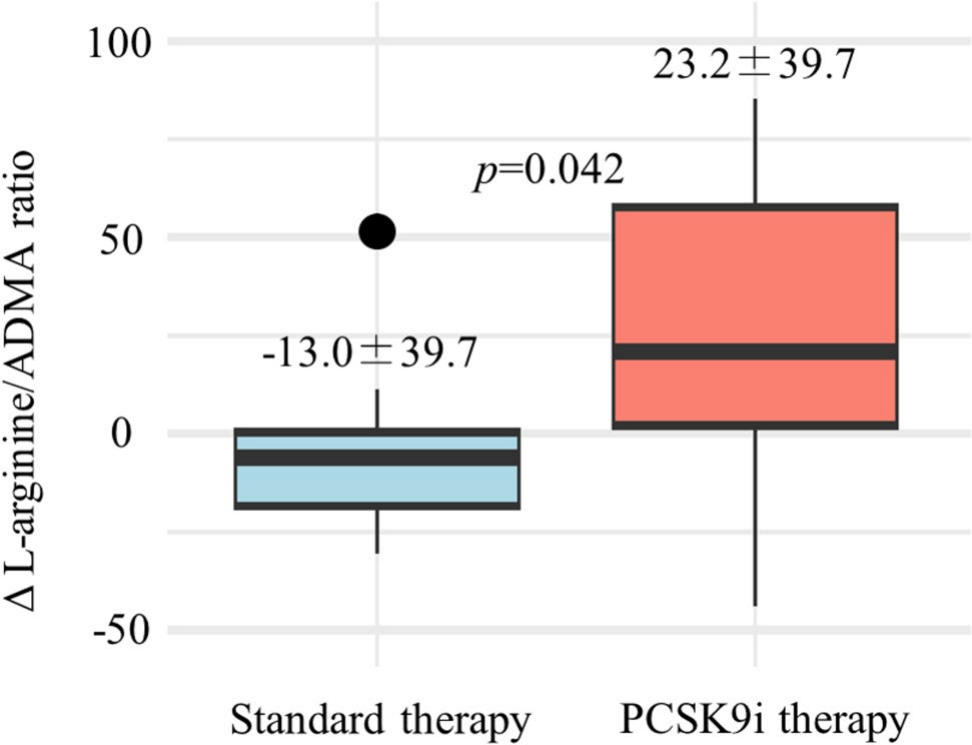
Changes in the L-arginine/asymmetric-dimethylarginine (ADMA) ratio (Δ L-arginine/ADMA ratio) for both the standard therapy and PCSK9 inhibitor (PCSK9i) groups between admission and discharge. Each boxplot illustrates the interquartile range (IQR) of the changes in TNF-α levels, with the median value highlighted. Whiskers extend from the box to the furthest data points that are within 1.5 times the IQR, denoting variability outside the central 50% of the data. Points beyond the whiskers are outliers, showing extreme L-arginine/ADMA ratio changes

**Fig. 4:**
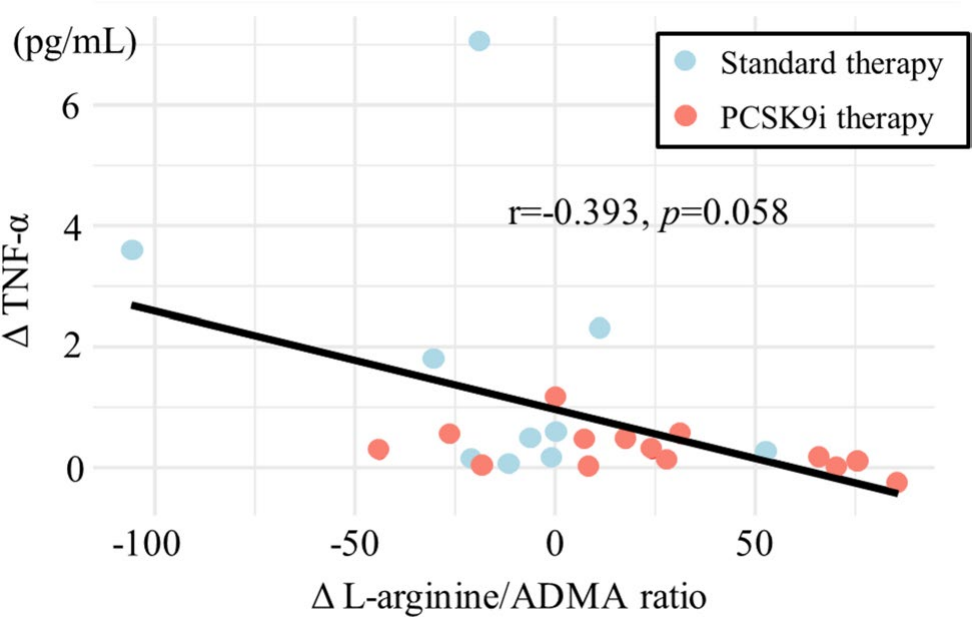
Correlation between the change in tumor necrosis factor-α (TNF-α) levels (Δ TNF-α) and the change in L-arginine/asymmetric dimethylarginine (ADMA) ratio (Δ L-arginine/ADMA ratio). A weak negative correlation was suggested between the change in TNF-α levels and the change in L-arginine/ADMA ratio, although not statistically significant (*r* = − 0.393, *p* = 0.058)

Similar to TNF-α, no significant difference was observed in the L-arginine/ADMA ratio at the time of admission between the two groups, with the standard therapy group at 123.7 ± 41.7 and the PCSK9 inhibitor group at 111.8 ± 34.4 (*p* = 0.451). In contrast, the L-arginine/ADMA ratio was higher in the PCSK9 inhibitor group than in the standard therapy group at discharge. The change in the L-arginine/ADMA ratio was − 13.0 ± 39.7 and 23.2 ± 39.7 in the standard therapy and PCSK9 inhibitor groups, respectively (*p* = 0.042) (Fig. 3). In a multiple linear regression analysis adjusted for sex, age, peak CK levels, and smoking, the L-arginine/ADMA ratio was also found to be increased in the PCSK9 inhibitor group (*p* = 0.018; *β* = 41.913, 95% CI, 10.337–73.491) (Table 3). Furthermore, a weak negative correlation was suggested between the change in TNF-α levels and the change in L-arginine/ADMA ratio, although not statistically significant (*r* = − 0.393, *p* = 0.058) (Fig. 4).

**Table 3:** Multiple regression analysis for the change in L-arginine/ADMA ratio

	Beta	Std. error	95% CI	*t*	*p* value
Constant	− 2.766	54.661	− 109.900 to 104.369	− 0.051	0.960
Sex (male)	− 8.751	26.100	− 61.667 to 44.164	− 0.324	0.750
Age (year)	− 0.216	0.721	− 1.628 to 1.197	− 0.299	0.768
maximal CK (U/I)	− 0.007	0.003	− 0.013 to − 0.001	− 2.446	0.025
Smoking	35.335	20.516	− 4.875 to 75.545	1.722	0.102
PCSK9i therapy	41.913	16.111	10.337 to 73.491	2.602	0.018

The change in the L-arginine/ADMA ratio was defined as the difference between the change in the L-arginine/ADMA ratio at discharge and admission. In a multiple linear regression analysis, with the change in L-arginine/ADMA ratio as the dependent variable and sex, age, maximal CK, smoking, and PCSK9i therapy as independent variables, PCSK9i therapy significantly increased the L-arginine/ADMA ratio

*ADMA* asymmetric-dimethylarginine; *maximal CK* maximal creatine kinase; *PCSK9i therapy* proprotein convertase subtilisin kexin 9 inhibitor therapy; *L-arginine/ADMA* L-arginine/asymmetric-dimethylarginine; *CI* confidence interval

## Discussion

The present study found that TNF-α levels in the PCSK9 inhibitor group were lower than those in the standard therapy group and that the L-arginine/ADMA ratio in the PCSK9 inhibitor group was higher than that of the standard therapy group. Moreover, we observed a negative correlation between the change in TNF-α levels and the change in L-arginine/ADMA ratio.

### PCSK9 inhibitor-mediated TNF-α suppression

This study demonstrated that PCSK9 inhibitor therapy administered in the early phase after AMI reduced reperfusion injury-related TNF-α levels [6]. Typically, TNF-α levels increase in the early phase after AMI due to the following mechanism. In the initial 24 h of reperfusion, the injured myocardium undergoes infiltration by neutrophils, monocytes, and lymphocytes, while resident mast cells release TNF-a, which initiates the cytokine cascade [19]. All cardiac cells express Toll-like receptors (TLR) 2–4 and TLR6, with TLR4-bearing cells activated via a TLR4 signal due to oxidative stress and the accumulation of activated complement during myocardial ischemia reperfusion, which triggers the onset and development of an inflammatory response [20]. TLR4 induction leads to a significant increase in proinflammatory cytokines, such as TNF-α, within the myocardium through the activation of nuclear factor-κB [21]. Therefore, in patients with AMI, the plasma levels of TNF-α and activated TLR4 are significantly higher than those in healthy individuals [6, 20–23]. PCSK9 can bind to the C-terminal domain of TLR4, thereby promoting the activation of the TLR4/NF-κB signaling pathway and subsequent secretion of inflammatory cytokines [24, 25]. Thus, we can infer that the PCSK9 inhibitor therapy can effectively suppress TNF-α production by inhibiting the TLR4/NF-κB signaling pathway.

Suppressing TNF-α production and activity in the early acute phase of myocardial infarction has been shown to improve cardiac function [26, 27]. While our study did not evaluate the progression of cardiac function, the use of PCSK-9 inhibitors in AMI has the potential to enhance cardiac function.

### PCSK9 inhibitor-mediated change in L-arginine/ADMA ratio and its correlation with the change observed in TNF-α levels

We demonstrated that PCSK9 inhibitor therapy not only improved the L-arginine/ADMA ratio during the acute phase after AMI but also mediated changes that correlated with the suppression of TNF-α levels. Myocardial infarction causes a decrease in L-arginine and an increase in ADMA that results in a reduction in the L-arginine/ADMA ratio, which reflects microvascular dysfunction [7, 8]. Inflammation reduces L-arginine levels by limiting its transport to endothelial cells through the y + transporter system [28]. Moreover, in human endothelial cells, TNF-α increases ADMA levels by inhibiting the activity of dimethylarginine dimethylaminohydrolase, a critical component of the ADMA metabolic pathway [28]. Consequently, this results in a negative correlation between TNF-α levels and the L-arginine/ADMA ratio.

PCSK9 expression in the ischemic heart has been well documented, and the administration of PCSK9 inhibitors in mice has been shown to reduce autophagy and infarct size [29]. Similarly, the present study found that PCSK9 inhibitor therapy in the early phase after AMI effectively mitigated reperfusion injury and microvascular dysfunction by suppressing TNF-α and improving the L-arginine/ADMA ratio. This, in turn, mitigates myocardial injury in AMI.

### Limitations

This study had several limitations. First, the study was conducted at a single facility, which might constrain the wider applicability of the results. Second, we did not assess the clinical outcomes of patients in this study, such as the incidence of heart failure or recurrent cardiovascular events. Consequently, it is essential to undertake further studies involving larger participant cohorts and extended periods of observation to ascertain the clinical relevance of our findings. Third, although we observed significant changes in TNF-α levels and the L-arginine/ADMA ratio, we did not directly evaluate the effects of the PCSK9 inhibitor therapy in the acute phase after AMI on microvascular function or left-ventricular remodeling. Hence, future studies should prioritize investigating the impact of PCSK9 inhibitors on coronary flow reserve, the index of microcirculatory resistance measured by pressure wire, and end-systolic volume assessed by echocardiography. This will provide a more comprehensive understanding of their cardioprotective effects following AMI. Finally, this study was not a prospective randomized trial, and the sample size was relatively small. These factors may affect the generalizability and statistical power of our findings. Therefore, larger, multicenter, randomized-controlled trials should be conducted in the future to validate and extend our findings.

## Conclusion

PCSK9 inhibitor therapy in the early phase after AMI suppressed TNF-α levels and improved the L-arginine/ADMA ratio. These findings highlight the potential pleiotropic effects of PCSK9 inhibitors in the management of patients with AMI.

## Data Availability

The datasets used and/or analysed during the current study are available from the corresponding author on reasonable request.
